# Differential Diagnosis of Chorea—HIV Infection Delays Diagnosis of Huntington’s Disease by Years

**DOI:** 10.3390/brainsci11060710

**Published:** 2021-05-27

**Authors:** Jannis Achenbach, Simon Faissner, Carsten Saft

**Affiliations:** Department of Neurology, Huntington Center North Rhine-Westphalia, Ruhr-University Bochum, St. Josef-Hospital Bochum, Gudrunstraße 56, 44791 Bochum, Germany; simon.faissner@rub.de (S.F.); carsten.saft@rub.de (C.S.)

**Keywords:** Huntington’s disease, human immunodeficiency virus infection, clinical expression, ENROLL-HD

## Abstract

Background: There is a broad range of potential differential diagnoses for chorea. Besides rare, inherited neurodegenerative diseases such as Huntington’s disease (HD) chorea can accompany basal ganglia disorders due to vasculitis or infections, e.g., with the human immunodeficiency virus (HIV). The clinical picture is complicated by the rare occurrence of HIV infection and HD. Methods: First, we present a case suffering simultaneously from HIV and HD (HIV/HD) focusing on clinical manifestation and disease onset. We investigated cross-sectional data regarding molecular genetic, motoric, cognitive, functional, and psychiatric disease manifestation of HIV/HD in comparison to motor-manifest HD patients without HIV infection (nonHIV/HD) in the largest cohort of HD patients worldwide using the registry study ENROLL-HD. Data were analyzed using ANCOVA analyses controlling for covariates of age and CAG repeat length between groups in IBM SPSS Statistics V.25. Results: The HD diagnosis in our case report was delayed by approximately nine years due to the false assumption that the HIV infection might have been the cause of chorea. Out of *n* = 21,116 participants in ENROLL-HD, we identified *n* = 10,125 motor-manifest HD patients. *n* = 23 male participants were classified as suffering from HIV infection as a comorbidity, compared to *n* = 4898 male non-HIV/HD patients. Except for age, with HIV/HD being significantly younger (*p* < 0.050), we observed no group differences regarding sociodemographic, genetic, educational, motoric, functional, and cognitive parameters. Male HIV/HD patients reported about a 5.3-year-earlier onset of HD symptoms noticed by themselves compared to non-HIV/HD (*p* < 0.050). Moreover, patients in the HIV/HD group had a longer diagnostic delay of 1.8 years between onset of symptoms and HD diagnosis and a longer time regarding assessment of first symptoms by the rater and judgement of the patient (all *p* < 0.050). Unexpectedly, HIV/HD patients showed less irritability in the Hospital Anxiety and Depression Scale (all *p* < 0.05). Conclusions: The HD diagnosis in HIV-infected male patients is secured with a diagnostic delay between first symptoms noticed by the patient and final diagnosis. Treating physicians therefore should be sensitized to think of potential alternative diagnoses in HIV-infected patients also afflicted by movement disorders, especially if there is evidence of subcortical atrophy and a history of hyperkinesia, even without a clear HD-family history. Those patients should be transferred for early genetic testing to avoid further unnecessary diagnostics and improve sociomedical care.

## 1. Introduction

The neurodegenerative Huntington’s disease (HD) is caused by a CAG-trinucleotide repeat expansion in the huntingtin gene (*HTT*), resulting in manifold heterogeneous motoric, psychiatric, and cognitive impairments [1]. Although there is a plethora of clinical symptoms, overarching theories of underlying pathomechanisms and heterogeneous interactions have not been fully understood until now. Manifold findings from preclinical and clinical studies investigated diverse mechanisms, trying to generate models about underlying HD pathomechanisms with the aim to identify potential therapeutic targets. The role of the huntingtin protein and interactions with binding proteins (e.g., ubiquitin-conjugating enzyme) and its role in different molecular processes, like nuclear inclusions and the impact on cell death, were identified [2,3]. Furthermore, the expanded polyglutamine in HD results in abnormal protein–protein interactions with mitochondrial dysfunction, excitotoxicity, and altered gene transcription, leading to reduced neuronal survival [4].

Recently, a strong link between neurodegenerative processes and neuroinflammation regarding increased cytokine formation, inflammatory response, and neurodegenerative processes has been identified [5]. Inflammation in neurodegenerative diseases is increasingly seen as both a contributor and product of neurodegenerative progression, fueling microglia-mediated or endotoxin-induced inflammation, hence substantially contributing to molecular processes of neurodegenerative diseases [6,7,8]. Inflammatory mechanisms of the innate and adaptive immune system are seen as major contributors for the pathomechanisms of HD [9,10]. However, the link between neurodegeneration, neuroinflammation, and the expression of the clinical disease pattern has not been fully understood until now. The usage of immunomodulating substances has elicited a positive effect in in vivo models of HD [11,12,13,14]. The recently conducted clinical trial LEGATO-HD, investigating the effect of laquinimod in HD, however, failed to reach the primary endpoint [15]. Along with Alzheimer’s disease and Parkinson’s disease, HD and other neurodegenerative disorders, neurovascular diseases, chronic pain conditions, diabetes, and also human immunodeficiency virus (HIV) infection are characterized by CNS immune cell infiltrations, activated microglia, increased production of cytokines, and oxidative stress, hypothesizing, at least in part, similarities in the underlying mechanisms of the interplay and possible connections between neuroinflammation and neurodegeneration [16,17,18,19,20].

The underlying pathomechanisms in HIV are accompanied by a decline of neuronal cell survival, resulting in increasing rates of neurodegeneration [21]. The release of chemokines in neurodegenerative diseases with increased microglia proliferation and inflammation is similarly described in HIV, leading to synapto-dendritic alterations and neuronal loss supporting cognitive and motoric impairment [22]. Systematic immune response influenced by metabolic activities of the gut microbiota–neuroinflammation axis has been discussed as a potential underlying mechanism for HIV [23,24]. Moreover, HIV infection can lead to heterogeneous clinical abnormalities ranging from encephalitis, myelitis, polyneuropathy, and infiltrative nerve lymphocytosis [25]. Critically, HIV and HD can manifest with a similar clinical picture, such as movement disorders with chorea and neuropsychiatric symptoms with alterations of mood [16].

Another critical manifestation of HIV infection relates to a decline of cognition, summarized under the term HIV-associated neurocognitive disorder (HAND), which can occur even though the viral load can efficiently be suppressed using highly active antiretroviral therapy (HAART) [26]. Pathomechanistically, HAND is induced by direct viral neurotoxicity and the interplay between HIV-infected monocytes and microglia, leading to neurodegeneration [18,27]. Despite treatment with HAART, about 50% of infected HIV patients are affected by HAND [26]; thus, identifying potential therapeutic targets is critically needed [28].

There are similarities between the clinical manifestations of HIV and HD. A spectrum of clinical disorders, especially with hyperkinetic movements such as chorea, dystonia, and myoclonus, is known in HIV and other viral infections caused by an infection of the CNS, especially the basal ganglia [29]. Hemiballism-hemichorea and tremor were reported as the most common hyperkinetic movement disorders in HIV, with 3% of all patients with HIV infection and 50% of patients with AIDS developing clinically relevant movement disorders [30]. Additionally, case reports have described hemichorea and choreoathetosis in patients with HIV, even as initial symptoms in patients with persisting low-level viremia [31,32,33]. A recent case series described eight patients suffering from HIV and HD, who had a nine year earlier disease onset of HD compared to other motor-manifest HD patients, potentially caused by a neurobiological link between the two diseases [34]. The authors hypothesized that combined effects of an HIV infection with changes induced by HD results in more pronounced molecular and neuropathologic changes with faster disease progression. 

To the best of our knowledge, cognitive, functional, and psychiatric disease manifestations of patients suffering from both HIV and HD have not yet been investigated so far. The evaluation of clinical characteristics potentially caused by effects of both diseases might help to get a better understanding about underlying neuropathologic mechanisms.

We first described a case report of a patient suffering from HIV and HD, treated in our Huntington Center. We then analyzed data derived from the global research platform ENROLL-HD to identify patients suffering from HD and coincident HIV and compared those to a large cohort of HD patients without HIV infection. 

## 2. Methods

### 2.1. A Case of HIV/HD

As a first approach, we present a case of HIV/HD from our Huntington Center focusing on clinical manifestation and disease onset in order to demonstrate difficulties in the diagnostic workup. This illustrative case was reflected in data of the ENROLL-HD registry study. The patient presented in the case report gave written informed consent for the publication of his case. 

### 2.2. ENROLL-HD Database: Inclusion and Exclusion Criteria

We then analyzed data from the global registry study ENROLL-HD [35]. ENROLL-HD is a global clinical research platform designed to facilitate clinical research in Huntington′s disease (HD). Core datasets are collected annually from all research participants as part of this multicenter longitudinal observational study. Data are monitored for quality and accuracy using a risk-based monitoring approach. All sites are required to obtain and maintain local ethical approval. Ethics approval was obtained by the local ethics committee of Ruhr University, Bochum (No. 4941-14). 

We analyzed the periodic dataset five (PDS-5) and included patients from category 3 (hdcat 3: motor-manifest HD), having a diagnostic confidence level of 4 (Diagconf 4: having unequivocal motor-manifest symptoms of HD/>99% confidence), a Unified Huntington′s Disease Rating Scale (UHDRS)—total motor score (TMS) >5, a genetically confirmed report with ≥36 cytosine-adenine-guanine (CAG) repeats in the huntingtin gene (*HTT)*, and an age >18 years. Then, comorbidities were analyzed, investigating participants suffering from “unspecified human immunodeficiency virus disease”, “asymptomatic human immunodeficiency virus infection status”, “acute HIV infection syndrome”, and “HIV disease resulting in other viral infections”. Since *n* = 23/25 patients with HIV/HD were male and the database of female patients with *n* = 2 was not sufficient to perform robust statistical analyses, further analysis only included male HIV/HD and male HD control patients. Antiretroviral therapy of HIV/HD was classified according to the international anatomical therapeutic chemical (ATC) classification of the World Health Organization (WHO).

### 2.3. Statistical Analyses

We analyzed group the means and standard deviations at baseline visit of study participants. Following this, we conducted univariate analysis of variance for sociodemographic data, genetics (CAG repeat length), education level (ISCED), and age. Motoric, functional, cognitive, psychiatric, and onset parameters were assessed using ANCOVA analysis, controlling for the co-variates of CAG repeat length and age between motor-manifest HIV/HD and non-HIV/HD using IBM SPSS Statistics, Version 25.0 (IBM Corp, Armonk, NY, USA).

## 3. Results

### 3.1. A Case Report: Hyperkinesia in Coinciding HIV Infection with HD—7–9 Years of Diagnostic Delay of HD 

**Figure 1:**
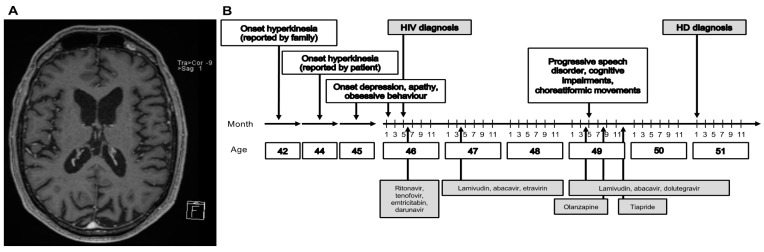
Case report of delayed HD diagnosis in an HIV-infected patient. (**A**) Magnetic resonance tomography (MRI) showing caudate atrophy at the age of 49. Multiplanar reformation (MPR) technique. (**B**) Clinical, diagnostic, and therapeutic features of the case report.

We report about a 53 year old Caucasian male patient. The patient was diagnosed with human immunodeficiency virus (HIV) infection at age 46 and treated in the local university hospital. Treatment of HIV infection following initial diagnosis for 7 months consisted of ritonavir (RTV)/tenofovir and emtricitabin (TVD)/darunavir (DRV), which was then changed, because of companion diseases, to lamivudin (3TC)/abacavir (ABC)/etravirin (ETV) (2 years), and was then, because of reported side effects, changed to dolutegravir/abacavir/lamivudin for more than 3 years. Three years after initial diagnosis, the patient presented with a progressive speech disorder with “hacked speech” and aphasia, as well as “involuntary convulsions” in the left corner of the mouth, noticed by the family. He had difficulties in concentration and psychomotor restlessness. Clinically, the patient had involuntary, choreatiformic movements. The patient was cachectic and treated with lamivudin/abacavir/dolutegravir (Th-cells 524/µL; HIV-PCR < 40 replicates (less than lower detection limit). MRI showed a distinct brain involution, clearly exceeding the age norm, but no evidence for any opportunistic infection (Figure 1A). A neuropsychologic workup documented a cognitive impairment with slowed cognition. A lumbar puncture revealed increased total protein (72 mg/dL) and a normal cell count. Electroneurography revealed a beginning primary sensomotoric demyelinating polyneuropathy. A hyperkinesia of unknown origin was diagnosed and the patient was treated with olanzapine 2.5 mg, without an effect on hyperkinesia. At the age of 49, the diagnosis “choreatiformic hyperkinesia potentially related to HIV-infection and HIV-encephalopathy with cognitive impairment” was made. Treatment was changed from olanzapine to tiapride 50 mg bid and later increased to 100 mg bid. The aforementioned diagnosis was confirmed in several outpatient consultations. At the age of 50, eight years after the onset of symptoms, following another comprehensive evaluation of the medical history, it became evident that the onset of hyperkinesia had been four years prior to HIV infection; therefore, the patient was tested for HD in a specialized outpatient department for movement disorders, which revealed a positive result with a CAG repeat length of 45 (tolerance +/−1) in the expanded allele of the huntingtin gene (Figure 1B). The family history revealed no relative with hyperkinesia or suspected movement disorders; an important finding described in about 8% of HD cases [1]. Of note, the father was alcoholic and died at the age of 45 years with “weight lost and emaciated within a few weeks”. 

Since the diagnosis of HD, the patient has performed weekly occupational and physical therapy. He has continued with psychotherapy, which was started at the age of 45. Following the diagnosis of HD, the patient received support by the local social-psychiatric service and a nursing service, he visited a local self-supporting group for people suffering from HD, and he started working in supervised nonprofit work. After diagnosis, he was able to gain more support for his social and financial situation, which improved his personal situation.

### 3.2. Clinical Characteristics of HIV/HD in the Global Research Study ENROLL-HD

**Figure 2:**
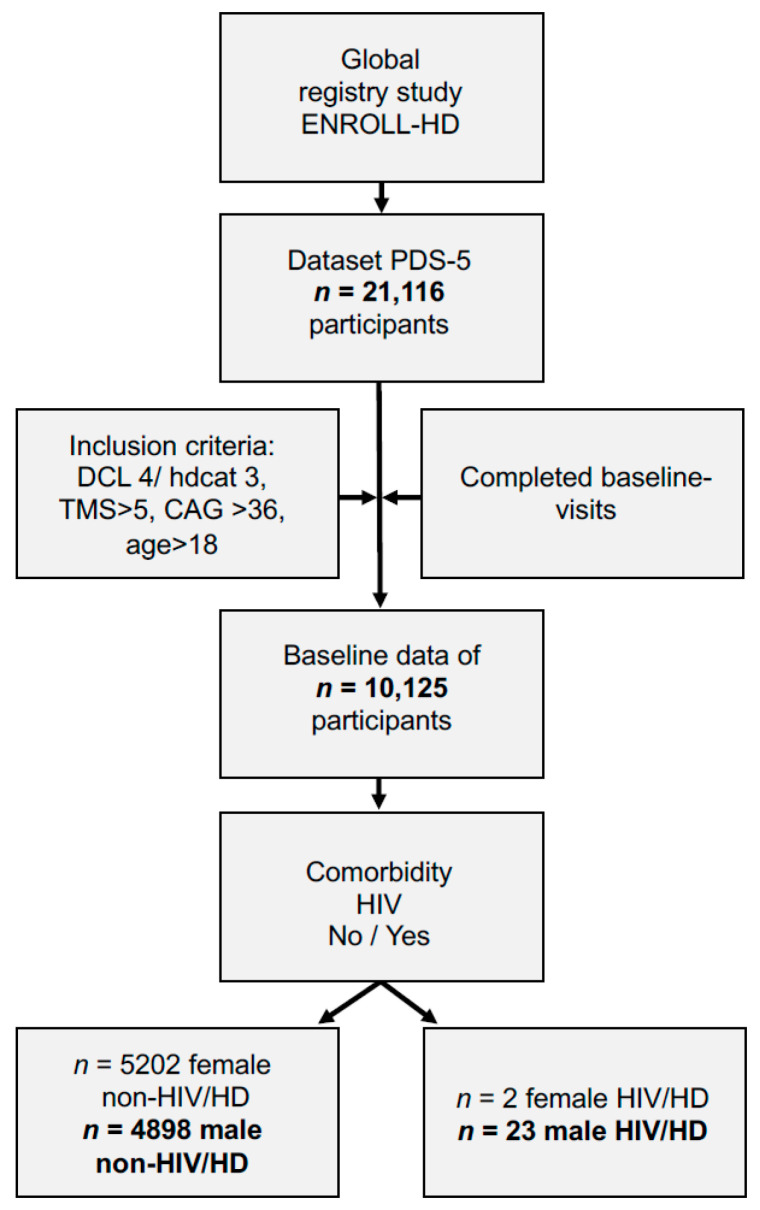
Data analysis of patients in ENROLL-HD regarding coinciding HIV infection. Abbreviations: CAG: cytosine-adenine-guanine repeat length; DCL: diagnostic confidence level; Hdcat: participant category; HD: Huntington´s disease; HIV: human immunodeficiency virus infection; PDS-5: periodic dataset five; TMS: total motor score.

Inspired by this case, we analyzed the global research study ENROLL-HD and identified *n* = 10,125 participants who met inclusion criteria out of *n* = 21,116 participants (Figure 2). Regarding analysis of comorbidities, we identified *n* = 25 patients with HIV/HD. Out of these participants *n* = 23 were male and *n* = 2 female. We statistically only analyzed data of male participants both in the HIV/HD group compared to *n* = 4898 other motor-manifest non-HIV infected male HD participants (non-HIV/HD) since there were not sufficient data for females in the HIV/HD group (*n* = 2). 

We analyzed moleculargenetic, demographic, motoric, and functional data of patients suffering from HIV/HD compared to HD. Male HIV/HD patients were significantly younger (*p* < 0.050) compared to HD. Moleculargenetic, educational, motoric, and functional parameters at baseline did not differ (Table 1). 

In addition to the analyzed 23 male patients, two female HIV/HD subjects aged 56 and 45 years were registered within the ENROLL-HD database. These women had a CAG repeat length of 43 and 46, respectively, in the expanded gene, and HD symptom onset was noticed by the patients at the age of 43 and 44 and diagnosis made at the age of 43 and 54 years. The two female patients were not included in further statistical analysis.

### 3.3. Symptom Onsets and Diagnostic Delay in Male HIV/HD

We then performed further analyses, controlling for the co-variables of age and CAG repeat length to exclude group differences of underlying pathophysiological parameters as potential confounders. We analyzed the age of onset of HD diagnosis and the age of first HD symptoms noticed by the patient, family, and rating of a medical professional. We found no differences between groups concerning age at HD diagnosis, symptom onset noticed by the family, and estimation by the clinical rater. However, HIV/HD patients reported about a 5.3-year-earlier onset of HD symptoms noticed by themselves compared to non-HIV/HD (*p* < 0.050). Moreover, we found that patients in the HIV/HD group had a longer diagnostic delay of 1.8 years between onset of symptoms and HD diagnosis compared to non-HIV/HD patients, as well as a longer time regarding assessment of first symptoms by the rater and judgement of the patient of 0.8 years (all *p* < 0.050; Table 2).

### 3.4. Cognitive Performance Did Not Differ between Male HIV/HD and Non-HIV/HD Groups

We also investigated the cognitive performance of respective groups. Cognition did not differ at baseline assessment in a test battery of seven cognitive tests, assessing cognition, verbal fluency, and flexibility of thinking among others (Table 3).

### 3.5. Male HIV/HD Participants Have Less Self-Reported Irritability Subscores Than Non-HIV/HD

We also analyzed psychiatric parameters and found no differences in psychiatric behavior between both groups reported by the clinical rater within the Problem Behaviors Assessment—Short (PBA-s) questionnaire. We then investigated subscores for depression, irritability/aggression, psychosis, apathy, and executive function. The self-reported assessment using the Hospital Anxiety and Depression Scale (HADS) questionnaire revealed that HIV/HD participants depicted significantly less irritability in the irritability subscore and a lower outward irritability subscore (all *p* < 0.05) (Table 4).

### 3.6. Pharmacotherapy in Male HIV/HD Participants

**Figure 3:**
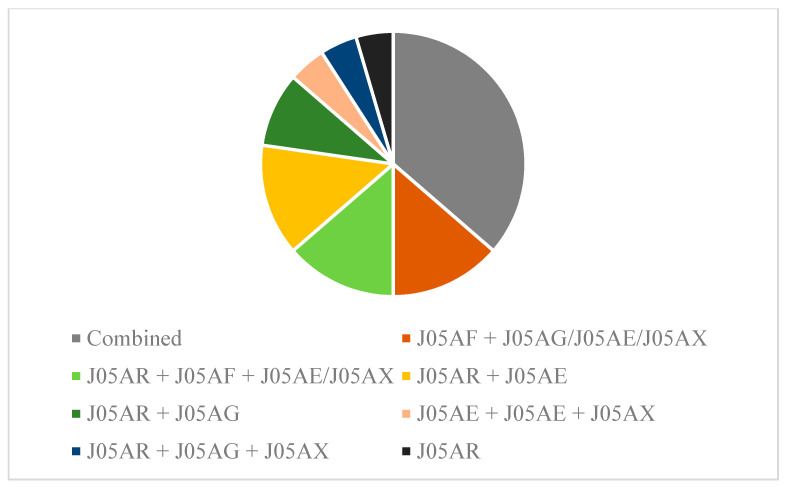
Pharmacotherapy and combinations in male HIV/HD. *n* = 8/23 patients received combined medications for HIV treatment, not further specified. *n* = 14 patients were treated with a combination therapy. Data are shown according to the WHO ATC codes (see methods section for further clarification).

In total *n* = 22/23 male HIV/HD participants were under continuous antiretroviral therapy due to the HIV infection. We identified five categories and agents classified as protease inhibitors (J05AE), nucleoside and nucleotide reverse transcriptase inhibitors (J05AF), non-nucleoside reverse transcriptase inhibitors (J05AG), antivirals for treatment of HIV, combinations (J05AR), and other antivirals (J05AX). No prophylactic intakes or one-time dosing for exposure were reported or included in the analysis. Eight patients were treated with a combination therapy classified as “antivirals for treatment of HIV infections, combinations, ingredient unspecified” on average since 1671 (± 1129 SEM) days prior to baseline assessment. Ten participants were treated with a combination, whereby nine of them received a combined medication additionally with nucleoside and nucleotide reverse transcriptase inhibitors, non-nucleoside reverse transcriptase inhibitors, protease inhibitors, or other antivirals (Figure 3).

## 4. Discussion

Using ENROLL-HD, which is the largest database of HD patients worldwide, we have shown that, in line with an inspiring case report, male patients suffering from HIV infection had a longer diagnostic delay of nearly two years between onset of symptoms reported by themselves and HD diagnosis compared to uninfected male HD patients. Unexpectedly, cognition was not more severely affected in patients suffering from both entities.

Differential diagnosis for chorea might be challenging in some cases [29]. We here reported about a case of combined HIV/HD who had a delay of 7–9 years between first symptoms noticed by the patient or family and the diagnosis of HD. Although manifold diagnostic approaches including lumbar puncture and MRI were performed, the diagnosis of HD was made because of the detailed and more accurate patient history with chronological assessments of observed hyperkinetic movement disorders late during the course of the disease [36]. 

HD is the most common reason for generalized hereditary chorea [37]. In order to avoid risks due to unnecessary diagnostic approaches, the initiation of a moleculargenetic testing of the mutant HTT might be appropriate as a first step if typical hyperkinetic movement disorders are observed [38,39]. We believe that this approach might be particularly justified in the case of a generalized chorea, while a hemichorea might be indicative of structural brain damage. The diagnosis might be more difficult if other movement disorders, such as myoclonus, dystonia, and hypokinetic rigidity are predominant or the family history is inconclusive, as in our case report [40,41,42,43,44]. A negative family history is reported in about 8–10% of HD patients [1]; hence, a negative family history does not exclude HD. In the case presented here, the father, who already died at the age of 45 years without having hyperkinetic movement disorders, was described as cachectic and alcohol dependent. One might hypothesize that the father did not reach the age for suffering of distinctive HD motor symptoms but might have been in the prodrominal phase of the disease, with anticipation leading to the extension of the mutant HD allele, which resulted in CAG-length-dependent earlier manifestation in our case [45,46]. This is supported by the fact that anticipation is inherited mostly through the male line and is accompanied with earlier age of symptomatic onset because of longer CAG repeats [47,48].

The diagnostic dilemma in the small cohort of patients suffering simultaneously from HIV and HD, investigated in the large cohort of ENROLL-HD participants, is complicated further, since also HIV can induce a broad spectrum of neurological complications including HIV-related movement disorders. [30]. Suffering from HIV and additionally from rare HD is obviously a challenging situation in terms of diagnosing both diseases. Conversely to the presented case report, Pereira et al. reported about a patient with a clinically based misdiagnosis of HD for four years suffering from severe progressive choreatiformic movement disorders because of a neurosyphilis and HIV [49]. The patient also did not have a positive HD family history but because of an intermediate CAG repeat length found in his father, anticipation with expansion of CAG repeat length in the son was suggested as a potential explanation [45,47]. The treating physicians therefore suggested immediate genetic testing for HD mutation, which would have avoided misdiagnosis [49]. The early diagnosis of HD is not only academic, since this might result, apart from improved medical treatment, in amelioration of sociomedical care and financial support [50], as seen in our case following HD diagnosis. 

Focusing on the large cohort of *n* = 4898 male motor-manifest non-HIV/HD patients from ENROLL-HD, we compared additionally *n* = 23 male HIV/HD patients with regard to clinical disease manifestation. Since only two out of 25 of the HIV/HD cohort in ENROLL-HD were female, we refrained from analyzing female patients in this cohort. Despite a younger age in male HIV/HD group, baseline data, including sociodemographic, genetic, motoric, and functional data, did not differ between groups. Concerning the age at HD diagnosis, no differences were reported in symptom onset noticed by the family and estimated by the clinical rater. However, male HIV/HD patients reported earlier onsets observed by themselves compared to HD. Likewise, we observed group differences, with longer periods between first symptoms noticed by the patient, HD diagnosis, and assessment of first symptoms by the rater (all *p* < 0.050). These findings are congruent with the described delay of 7–9 years between first symptoms noticed by the patient and HD diagnosis in the presented case report. In particular, the role of the HIV physician regarding the detection of HIV-related complications is known to be essential for the outcome [51]. Optimal treatment is complicated since HIV patients are suffering from HIV-related stigma even by healthcare providers, reducing patients’ quality of life [52,53]. Hence, it might be speculated that symptoms observed by the physician or reported by the patient might falsely have been attributed to the HIV infection, therefore potentially neglecting other differential diagnoses. Perceived stigma was also reported in HD, negatively impacting on quality of life [54], complicating the situation in patients afflicted by both diseases even further. 

Remarkably, we observed even less psychiatric symptoms in male HIV/HD participants going along with lower irritability and outward irritability subscores compared to non-HIV/HD male patients. This is contradictory to reports documenting that HIV/AIDS is associated with lower health-related quality of life (HRQoL), worse physical health and mental health status [55]. One might hypothesize that patients with HIV in combination with HD, which is associated with severe behavioral and psychiatric alterations, might lead to an aggravation of psychiatric symptoms [48]—a hypothesis not supported by our data. 

A high prevalence of HAND in HIV with predominant subcortical dementia (HIV-associated dementia: HAD) has been repeatedly described, even though simultaneously effective antiretroviral treatments lower the incidence of other opportunistic infections [56,57,58,59]. The reasons for cognitive impairment and a progress of decline in HIV patients are incompletely understood [58]. Concerning the pathobiology, HIV proteins are described as “virotoxins”, inducing neuronal cell loss and neuropathogenesis of HIV dementia [60]. Moreover, the infection of microglia and the formation of multinucleated giant cells, consisting of the latter and infected monocytes, lead to neuronal damage due to the release of inflammatory mediators [61,62]. Combined effects on pathobiology of HIV infection and HD were postulated to result in earlier onset of motoric HD symptoms [34]. Although HIV/HD participants appeared to be slightly younger regarding their medium age at HD diagnosis (47.3; SD 11.4), if compared to non-HIV/HD participants (49.3; SD 12.7), our data did not show significant differences between groups (*p =* 0.603). Additionally, we compared cognitive, functional, and psychiatric parameters of disease manifestation in male HIV/HD patients compared to the large cohort of motor-manifest male non-HIV/HD patients. Remarkably, no significant differences were detected using a large test battery. Thus, we could not confirm that a presumed aggravation of pathobiological molecular changes might negatively influence cognition in patients suffering from both entities.

To avoid a potential impact of diverse fundamental sociodemographic and genetic parameters, we controlled for age and CAG repeat length between groups, since age in particular has a negative impact on cognition [63]. Regarding the cognitive test battery, slight decreases were observed in all tests compared to population-based norms, identifying slight cognitive impairments in both groups. Both groups performed slightly under the norm of a mid-age population in the MMSE [63]. 

Combined antiretroviral therapy (cART) is seen as state of the art for the treatment of HIV [64,65]. In total, 95% of HIV-infected patients in our cohort were treated with a standard antiretroviral combination regimen, including the whole spectrum of cART therapy. The use of state-of-the-art antiretroviral treatment strategies is a potential explanation as to why functional and motoric disease manifestation did not differ between infected and uninfected patients. Motor signs with gait disturbances or impaired manual dexterity were identified as less common in HIV since cART strategies were implemented [56]. The use of antiretrovirals with high CNS penetration effectiveness in patients with HAND [66] has, however, been discussed, controversially showing both beneficial [67] and detrimental [68] effects due to potential neurotoxic effects of cART. To assess aspects of combined neuropathologic changes in HIV/HD in more detail, the analysis of tissue and the implementation of neuroimaging would be necessary. 

There are some limitations that need to be stated. First, the group of male HIV/HD was quite small. For a better understanding, a larger cohort would be more appropriate. We, however, believe that assessing the worldwide registry study ENROLL-HD with more than 21,100 participants currently is the best way to identify patients with combined HIV and HD. Moreover, since all but two of the HIV-infected patients were male, no conclusions regarding characteristics of females suffering from HIV/HD can be drawn, which is a clear limitation. Therefore further studies on females suffering simultaneously from HIV/HD are necessary to confirm our findings in females. Another limitation is the lack of imaging data, which are not included in the ENROLL-HD dataset. 

## 5. Conclusions

In summary, we have shown that there is a considerable diagnostic delay of years between the onset of clinical signs and HD diagnosis in male patients suffering from both HIV and HD, potentially explained by a blind spot in treating physicians who attribute new symptoms to the known HIV infection. Treating physicians therefore should be sensitized to think of potential alternative diagnoses, especially if there is evidence of subcortical atrophy and a history of more generalized hyperkinesia, even without a clear family history of HD. Those patients should be transferred for early genetic testing to avoid further unnecessary diagnostics and improve sociomedical care.

## Figures and Tables

**Table 1:** Baseline data of male HIV/HD and non-HIV/HD including sociodemographic, genetic, motoric, and functional data.

	HIV/HD *n* = 23	Non-HIV/HD *n* = 4898	*F*	*p*	Part. Eta^2^
Age (y); M (SD) (co-variate CAG)	50.5 (10.0)	53.3 (12.7)	4.048	0.044 *	0.001
CAG high (co-variate age)	43.7 (4.0)	44.0 (4.0)	3.015	0.083	0.001
ISCED +	3.9 (1.5)	3.4 (1.2)	2.210	0.137	0.000
Motoric UHDRS TMS; M (SD) #	33.3 (19.0)	37.6 (20.5)	0.026	0.871	0.000
TFC; M (SD) +	9.0 (3.3)	8.4 (3.6) (*n* = 4845)	0.005	0.945	0.000
IS; M (SD) +	79.6 (18.0)	77.9 (18.3) (*n* = 4889)	0.120	0.729	0.000

Motoric and functional data were analyzed with the co-variates of age and CAG repeat length. Significance is depicted as: * = *p* < 0.05. Abbreviations: M: mean; SD: standard deviation; *p*: *p* value; *F*: *F* value; Part Eta^2^: effect size; UHDRS: Unified Huntington′s Disease Rating Scale; CAG: cytosine-adenine-guanine repeat length; ISCED: educational level; TMS: total motor score; TFC: total functional capacity; IS: independence scale; +: higher scores = better performance; #: higher scores = more impairment.

**Table 2:** Symptom onset and diagnosis of male HIV/HD and non-HIV/HD.

	HIV/HD *n* = 23	Non-HIV/HD *n* = 4898	*F*	*p*	Part. Eta^2^
Hddiagn (y)	47.3 (11.4) (*n* = 22)	49.3 (12.7) (*n* = 4780)	0.271	0.603	0.000
Sxrater (y)	42.2 (9.0) (*n* = 20)	45.6 (12.4) (*n* = 4593)	0.071	0.790	0.000
Sxfam (y)	42.7 (11.7) (*n* = 19)	45.9 (12.6) (*n* = 4502)	0.479	0.489	0.000
Sxsubj (y)	41.0 (11.1) (*n* = 19)	46.3 (12.4) (*n* = 4841)	3.906	0.048 *	0.001
DIFF Sx Rater- Sx Subject	1.4 (6.4) (*n* = 18)	0.6 (3.3) (*n* = 4570)	6.723	0.010 *	0.001
DIFF HD Diagn- Sx Subject	4.8 (8.1) (*n* = 18)	3.0 (4.4) (*n* = 4747)	3.867	0.049 *	0.001
DIFF HD Diagn- Sx Rater	3.0 (5.6) (*n* = 19)	3.6 (4.3) (*n* = 4533)	0.150	0.699	0.000
DIFF HD Diagn- Sx Fam	4.0 (7.2) (*n* = 18)	3.6 (4.6) (*n* = 4451)	0.368	0.544	0.000

Data were analyzed using ANOVA analysis, controlling for the co-variates of age, CAG repeat length. Significance is depicted as: * = *p* < 0.05. Abbreviations: M: mean; SD: standard deviation; *p*: *p* value; *F*: *F* value; Part Eta^2^: effect size; Hddiagn: Huntington´s disease diagnosed; Sxrater: rater′s estimate of symptom onset; Sxfam: family’s estimate of symptom onset; Sxsubj: subject’s estimate of symptom onset; y: years; DIFF: difference in years.

**Table 3:** Cognitive data of male HIV/HD and non-HIV/HD.

	HIV/HD *n* = 23	Non-HIV/HD *n* = 4898	*F*	*p*	Part. Eta^2^
SDMT; M (SD) +	24.9 (11.2) (*n* = 21)	23.8 (11.3) (*n* = 4281)	0.009	0.925	0.000
Verfct; M (SD) +	14.4 (5.5) (*n* = 22)	12.1 (5.5) (*n* = 4648)	1.662	0.197	0.000
SCNT; M (SD) +	45.4 (14.0) (*n* = 21)	42.4 (16.2) (*n* = 4538)	0.146	0.703	0.000
SWRT; M (SD) +	59.9 (23.4) (*n* = 21)	56.8 (21.4 (*n* = 4523)	0.036	0.850	0.000
SIT; M (SD) +	25.0 (10.3) (*n* = 20)	23.6 (10.8) (*n* = 3922)	0.001	0.973	0.000
Trla #	66.8 (42.3) (*n* =16)	70.9 (50.4) (*n* = 3273)	0.041	0.839	0.000
MMSE +	26.8 (2.7) (*n* = 16)	25.0 (4.2) (*n* = 3065)	1.405	0.236	0.000

Data were analyzed using ANOVA-analysis controlling for the co-variates of CAG repeat length and age. Abbreviations: M: mean; SD: standard deviation; *p*: *p* value; *F*: *F* value; Part Eta^2^: effect size; SDMT: symbol digit modality test; Verfct: verbal fluency test; SCNT: Stroop color naming test; SWRT: Stroop word reading test; SIT: Stroop interference test; Trla: trailmaking test; MMSE: mini mental state examination; +: higher scores = better performance; #: higher scores = more impairment.

**Table 4:** Psychiatric data of male HIV/HD compared to non-HIV/HD.

	HIV/HD *n* = 23	Non-HIV/HD *n* = 4898	*F*	*p*	Part. Eta^2^
PBA_depscore #	4.4 (6.2)	4.6 (6.1)	0.163	0.686	0.000
Irascore #	1.7 (2.9)	3.4 (5.2)	2.820	0.093	0.001
Psyscore #	0.3 (1.2)	0.3 (1.8)	0.035	0.851	0.000
Aptscore #	1.6 (3.0)	3.5 (4.5)	3.164	0.075	0.001
Exfscore #	1.3 (2.6)	3.6 (5.5)	3.568	0.059	0.001
Hads_anxscore #	2.7 (4.1)	3.2 (4.1)	0.657	0.418	0.000
Depscore #	2.6 (3.9)	3.4 (4.3)	0.966	0.326	0.000
Irrscore #	1.5 (2.7)	3.2 (4.4)	4.633	0.031 *	0.001
Outscore #	0.9 (1.4)	2.0 (2.7)	4.563	0.033 *	0.001
Inwscore #	0.6 (1.5)	1.3 (2.2)	3.209	0.073	0.001

Data were analyzed using ANOVA-analysis controlling for the co-variates of age and CAG repeat length. Significance is depicted as: * = *p* < 0.05. Abbreviations: M: mean; SD: standard deviation; *p*: *p* value; *F*: *F* value; Part Eta^2^: effect size; PBA_depscore: Problem Behaviours Assessment—Short Depression; Irascore: irritability/aggression; Psyscore: psychosis; Aptscore: apathy; Exfscore: executive function; Hads_anxscore: Hospital Anxiety and Depression Scale anxiety subscore; depscore: depression subscore; Irrscore: irritability subscore; outscore: outward irritability subscore; Inwscore: inward irritability subscore; +: higher scores = better performance; #: higher scores = more impairment.

## Data Availability

The data that support the findings of this study are available from the corresponding author upon reasonable request.
